# Is gout a risk equivalent to diabetes for stroke and myocardial infarction? A retrospective claims database study

**DOI:** 10.1186/s13075-017-1427-5

**Published:** 2017-10-17

**Authors:** Jasvinder A. Singh, Rekha Ramachandaran, Shaohua Yu, Shuo Yang, Fenglong Xie, Huifeng Yun, Jie Zhang, Jeffrey R. Curtis

**Affiliations:** 1 Medicine Service, VA Medical Center, 700 South 19th Street, Birmingham, AL 35233 USA; 2 Department of Medicine, University of Alabama at Birmingham School of Medicine, 510 20th Street South, Faculty Office Tower 805B, Birmingham, AL 35294-0022 USA; 3 Division of Epidemiology, University of Alabama at Birmingham School of Public Health, 1720 Second Avenue South, Birmingham, AL 35294-0022 USA; 4 University of Alabama at Birmingham, 510 20th Street South, Faculty Office Tower 805B, Birmingham, AL 35294 USA

**Keywords:** Gout, Diabetes, Stroke, Myocardial infarction, Risk equivalent

## Abstract

**Background:**

Gout is a risk factor for cardiovascular disease, but associations with specific cardiovascular outcomes, myocardial infarction (MI), and stroke are unclear. Our objective in the present study was to assess whether gout is as strong a risk factor as diabetes mellitus (DM) for incident MI and incident stroke.

**Methods:**

In this retrospective study, we used U.S. claims data from 2007 to 2010 that included a mix of private and public health plans. Four mutually exclusive cohorts were identified: (1) DM only, (2) gout only, (3) gout and DM, and (4) neither gout nor DM. Outcomes were acute MI or stroke with hospitalization. We compared the age- and sex-specific rates of incident MI and stroke across the four cohorts and assessed multivariable-adjusted HRs.

**Results:**

In this study, 232,592 patients had DM, 71,755 had gout, 23,261 had both, and 1,010,893 had neither. The incidence of acute MI was lowest in patients with neither gout nor DM, followed by patients with gout alone, DM alone, and both. Among men >80 years of age, the respective rates/1000 person-years were 14.6, 25.4, 27.7, and 37.4. Similar trends were noted for stroke and in women. Compared with DM only, gout was associated with a significantly lower adjusted HR of incident MI (HR 0.81, 95% CI 0.76–0.87) but a similar risk of stroke (HR 1.02, 95% CI 0.95–1.10). Compared with patients with DM only, patients with both gout and DM had higher HRs for incident MI and stroke (respectively, HR 1.35, 95% CI 1.25–1.47; HR 1.42, 95% CI 1.29–1.56).

**Conclusions:**

Gout is a risk equivalent to DM for incident stroke but not for incident MI. Having both gout and DM confers incremental risk compared with DM alone for both incident MI and stroke.

**Electronic supplementary material:**

The online version of this article (doi:10.1186/s13075-017-1427-5) contains supplementary material, which is available to authorized users.

## Background

Gout is the most common inflammatory arthritis in adults [1]. Gout is a risk factor for cardiovascular disease [2], cardiovascular mortality [3], and all-cause mortality [3, 4]. However, associations with specific cardiovascular outcomes are unclear. Gout was associated with a higher risk of incident myocardial infarction (MI) by 1.23- to 1.82-fold in some studies [5–8], but it was not associated with higher risk in others [9, 10]. The association with stroke is also unclear. Researchers in a U.K. study found that the risk of incident stroke was 1.71 times higher in patients with gout than in the general population [7]. However, their study included only hospitalized patients, who have more severe gout and/or higher comorbidity burden than the general population, thereby limiting the generalizability of their findings [7]. Other limitations of previous studies were that adjustment for key cardiovascular risk factors was not performed in studies of MI [5, 7] and stroke [7], which raises a serious concern for significant confounding. Detailed analyses by age and sex (risk varies by these factors) were performed in one study [7], which did not control for cardiovascular risk factors such as hypertension and hyperlipidemia.

One mechanism of increased cardiovascular risk with gout may be due to hyperuricemia, a central abnormality in gout. Authors of systematic reviews of observational studies have found hyperuricemia by itself (even in the absence of gout) to be associated with a 1.47 times higher risk of stroke [11] and a 1.34 times higher risk of coronary heart disease (CHD) [12]. Hyperuricemia and associated mechanisms may partially mediate increased cardiovascular risk in patients with gout [6, 11–14].

Diabetes also is a well-known risk factor for cardiovascular disease. The National Cholesterol Education Program Expert Panel on Detection, Evaluation, and Treatment of High Blood Cholesterol in Adults (Adult Treatment Panel III) labeled diabetes as a cardiovascular risk equivalent, such that lipid management recommendations are similar for patients with diabetes and patients with CHD [15]. Despite being known as a cardiovascular risk factor, it is not known whether gout is a cardiovascular risk equivalent [16] similar to diabetes. Therefore, the objectives of the present study were (1) to assess whether gout is a cardiovascular risk equivalent for incident MI and stroke similar to diabetes, (2) to estimate the increased cardiovascular risk with gout for women and men in various age groups, and (3) to examine if gout is additive to diabetes for cardiovascular risk.

## Methods

We report study methods and results as recommended by the Strengthening the Reporting of Observational Studies in Epidemiology (STROBE) statement [17]. The institutional review board at the University of Alabama at Birmingham approved the study.

### Study cohort and participants

We conducted a retrospective cohort study using the Multi-Payer Claims Database (MPCD) for 2007–2010. This database linked health plan data from national commercial and governmental insurance plans, representing beneficiaries with United Healthcare, Medicare, and/or Medicaid coverage during 2007–2010. The MPCD contains patients’ demographic and insurance coverage information from enrollment files, claims for healthcare (inpatient and outpatient) services, and prescription medications. Participants were eligible if they had ≥ 12 months of continuous medical and pharmacy coverage to ensure a complete claims history (baseline period). Eligible patients were categorized into four mutually exclusive cohorts: (1) diabetes only, (2) gout only, (3) gout and diabetes, and (4) neither gout nor diabetes. There were no age restrictions on the dataset.

### Exposure of interest

The study exposure of interest was a diagnosis of gout, ascertained by the presence of International Classification of Diseases, Ninth Revision, Clinical Modification (ICD-9-CM) code 274.xx for gout, during an inpatient visit or at two outpatient visits with a physician encounter ≥ 7 days apart, which has been shown to have high positive predictive value of > 90% [18]. Diabetes was ascertained by the presence of two ICD-9-CM codes of 250.xx ≥ 7 days but ≤ 365 days apart and the use of a diabetes-specific medication, including insulin preparations, biguanide (metformin), thiazolidinediones (pioglitazone, rosiglitazone), sulfonylureas (first generation: tolbutamide, tolazamide, chlorpropamide, acetohexamide; second generation: glipizide, glyburide, glimepiride), sulfonylurea secretagogues (nateglinide, repaglinide), α-glucosidase inhibitors (acarbose, miglitol), injectable incretin mimetics (exenatide, liraglutide, taspoglutide, sitagliptin, saxagliptin, linagliptin), injectable amylin analogue (pramlintide), glucagon/dextrose, and drug combinations (e.g., glipizide/metformin) [19], a validated definition with high positive predictive value > 90% [20–22]. An individual was considered to have diabetes (diabetes cohort-eligible) the day he or she satisfied all these criteria. The index disease when a patient was considered to have diabetes and gout was the more recent of the two dates when a person became gout- and/or diabetes-eligible. Study follow-up started when the later of the following two conditions was met: disease eligibility (gout and diabetes) or 12 months of continuous coverage. Study follow-up ended when either of the two outcomes (described below) occurred; when the patient lost medical or pharmacy coverage; when the patient died; or on December 31, 2010.

### Study outcomes

The study outcomes were the incident hospitalized MI and the incident hospitalized stroke. Incident MI and incident stroke were defined as at least one inpatient claim with hospital discharge with ICD-9-CM codes for acute MI or stroke, respectively (*see* Additional file 1: Appendix 1 for more detail), and at least one night of inpatient stay, unless the patient died during the index hospitalization. These approaches of identifying patients with incident MI and stroke are valid with a high positive predictive value > 90% [22–24]. We excluded patients with any of the following conditions in the baseline period of 12 months prior to the start of follow-up (diagnoses of gout and diabetes and 12 months of continuous coverage met) to ensure that we were capturing incident MI and incident stroke: MI (410.xx or 412.xx), stroke (430–438) and heart disease (410–414, 428.xx, and 429.2x).

### Covariates and confounders

Study covariates included demographics (age, sex, race/ethnicity); cardiovascular risk factors other than diabetes (hypertension, hyperlipidemia); and other medical comorbidities, including chronic obstructive pulmonary disease (COPD), peripheral vascular disease (PVD), renal failure, and autoimmune diseases selected on the basis of their potential association with the outcome or common occurrence in the population of interest (*see* Additional file 1: Appendix 1 for more detail). Comorbidities and risk factors were defined on the basis of the presence of ICD-9-CM codes for each condition. For hyperlipidemia, we considered patients with either an ICD-9-CM code for hyperlipidemia or the use of a statin drug, because hyperlipidemia is an often undercoded diagnosis [25]. Age was categorized in 5-year age groups, and race/ethnicity was categorized as Asian, Hispanic, black, white, other, or missing. Other variables were categorized as follows: hypertension (yes/no), hyperlipidemia (yes/no), PVD (yes/no), COPD (yes/no), chronic kidney disease (yes/no), and autoimmune disease (yes/no). Smoking status, fasting lipid levels, and blood pressure readings (in millimeters of mercury) were not available in this database.

### Statistical analyses

We calculated summary statistics as proportions (in percent). We calculated rates of incident MI or incident stroke per 1000 person-years of exposure. We used multivariable-adjusted Cox regression analyses to assess the HR of incident MI and incident stroke. We performed unadjusted regression analyses to obtain crude estimates. Age-adjusted and multivariable-adjusted analyses were performed next to account for age (the most significant cardiovascular risk factor) vs. several other cardiovascular risk factors (hypertension, hyperlipidemia) and common comorbidity confounders (COPD). We performed statistical analyses using SAS version 9.3 software (SAS Institute, Cary, NC, USA). The proportional hazards assumption holds true for the analyses reported. A *p* value < 0.05 was considered significant. We performed sensitivity analyses by additionally adjusting for the following factors that can potentially impact the risk of MI/stroke: (1) nonsteroidal anti-inflammatory drugs (NSAIDs; associated increased risk) and medications used for the treatment of heart disease (risk reduction); and (2) autoimmune diseases, owing to associated chronic inflammation.

## Results

### Cohort characteristics

The overall cohort consisted of individuals contributing 1,338,501 observed study periods. Demographic and clinical characteristics of the four cohorts (diabetes only, gout only, both diabetes and gout, neither diabetes nor gout) are shown in Table 1. Patients with gout, diabetes, or both were slightly older, more likely to be men, and had higher rates of comorbidities than the cohort with neither condition (Table 1). Patients with diabetes only had a higher rate of comorbidities than those without either condition and a lower rate than patients with both gout and diabetes (Table 1). Characteristics of patients with and without incident MI as well as with and without incident stroke are shown in Table 2. Those with incident MI or incident stroke were older, more likely to be white, and more likely to have any of the comorbidities of interest than patients without incident MI or incident stroke (Table 2). The median (25th–75th percentile) follow-up was 427 days (245–988).

**Table 1:** Patient characteristics

	DM only (*n* = 232,592)	Gout only (*n* = 71,755)	DM and gout (*n* = 23,261)	Neither DM nor gout (*n* = 1,010,893)
Male sex	97,041	48,305	13,192	395,608
	(41.7%)	(67.3%)	(56.7%)	(39.1%)
Age group, years				
≤ 50	41,844	7910	1616	395,186
	(18.0%)	(11.0%)	(7.0%)	(39.1%)
51–60	43,982	7589	3057	125,502
	(18.9%)	(10.6%)	(13.1%)	(12.4%)
61–65	30,762	6268	2611	78,983
	(13.2%)	(8.7%)	(11.2%)	(7.8%)
66–70	40,974	14,044	5066	121,976
	(17.6%)	(19.6%)	(21.8%)	(12.1%)
71–75	30,601	12,055	4392	97,567
	(13.2%)	(16.8%)	(18.9%)	(9.7%)
76–80	22,807	10,386	3227	82,298
	(9.8%)	(14.5%)	(13.9%)	(8.1%)
> 80	21,620	13,501	3292	108,785
	(9.3%)	(18.8%)	(14.2%)	(10.8%)
Race				
Asian	6533	3270	970	24,744
	(2.8%)	(4.6%)	(4.2%)	(2.5%)
Black	34,551	10,358	4548	118,867
	(14.9%)	(14.4%)	(19.6%)	(11.8%)
Hispanic	14,100	1938	557	70,382
	(6.1%)	(2.7%)	(2.4%)	(7.0%)
Other	4199	934	470	11,254
	(1.8%)	(1.3%)	(2.0%)	(1.1%)
Missing	27,372	6418	1258	222,928
	(11.8%)	(8.9%)	(5.4%)	(22.1%)
White	145,837	48,837	15,458	562,718
	(62.7%)	(68.1%)	(66.5%)	(55.7%)
Comorbidities				
Hypertension	168,155	47,446	19,556	273,828
	(72.3%)	(66.1%)	(84.1%)	(27.1%)
COPD	20,835	5837	2376	50,610
	(9.0%)	(8.1%)	(10.2%)	(5.0%)
Chronic kidney disease	20,078	9366	5641	22,791
	(8.6%)	(13.1%)	(24.3%)	(2.3%)
Peripheral vascular disease	14,362	3850	1997	22,078
	(6.2%)	(5.4%)	(8.6%)	(2.2%)
Hyperlipidemia	168,964	36,296	18,066	262,591
	(72.6%)	(50.6%)	(77.7%)	(26.0%)

*Abbreviations: DM* Diabetes mellitus, *COPD* Chronic obstructive pulmonary disease

Hyperlipidemia is defined as a presence of a diagnostic code for hypercholesterolemia or statin use. Diagnostic codes for comorbidities are provided in Additional file 1: Appendix 1

**Table 2:** Characteristics of patients with incident myocardial infarction or incident stroke

	No MI (*n* = 1,325,037)	Incident MI (*n* = 13,464)	No stroke (*n* = 1,328,015)	Incident stroke (*n* = 10,486)
Male sex	548,447	5699	550,342	3804
	(41.4%)	(42.3%)	(41.4%)	(36.3%)
Age group, years				
≤ 50	445,916	640	446,085	471
	(33.7%)	(4.8%)	(33.6%)	(4.5%)
51–60	179,005	1125	179,348	782
	(13.5%)	(8.4%)	(13.5%)	(7.5%)
61–65	117,717	907	117,917	707
	(8.9%)	(6.7%)	(8.9%)	(6.7%)
66–70	179,931	2129	180,550	1510
	(13.6%)	(15.8%)	(13.6%)	(14.4%)
71–75	142,369	2246	142,889	1726
	(10.7%)	(16.7%)	(10.8%)	(16.5%)
76–80	116,375	2343	116,781	1937
	(8.8%)	(17.4%)	(8.8%)	(18.5%)
> 80	143,125	4073	143,846	3352
	(10.8%)	(30.3%)	(10.8%)	(32.0%)
Race				
Asian	35,218	299	35,269	248
	(2.7%)	(2.2%)	(2.7%)	(2.4%)
Black	166,744	1580	166,617	1707
	(12.6%)	(11.7%)	(12.6%)	(16.3%)
Hispanic	86,542	435	86,607	370
	(6.5%)	(3.2%)	(6.5%)	(3.5%)
Other	16,688	169	16,699	158
	(1.3%)	(1.3%)	(1.3%)	(1.5%)
Missing	257,540	436	257,627	349
	(19.4%)	(3.2%)	(19.4%)	(3.3%)
White	762,305	10,545	765,196	7654
	(57.5%)	(78.3%)	(57.6%)	(73.0%)
Comorbidities				
Hypertension	500,421	8564	502,233	6752
	(37.8%)	(63.6%)	(37.8%)	(64.4%)
COPD	77,833	1825	78,533	1125
	(5.9%)	(13.6%)	(5.9%)	(10.7%)
Chronic kidney disease	56,131	1745	56,741	1135
	(4.2%)	(13.0%)	(4.3%)	(10.8%)
Peripheral vascular disease	40,919	1368	41,446	841
	(3.1%)	(10.2%)	(3.1%)	(8.0%)
Hyperlipidemia	478,544	7373	480,711	5206
	(36.1%)	(54.8%)	(36.2%)	(49.7%)

*Abbreviations: MI* Myocardial infarction, *COPD* Chronic obstructive pulmonary disease

Data were analyzed after removing the patients with baseline International Classification of Diseases, Ninth Revision, Clinical Modification, codes of 410, 412, 430–438, 428.xx, and 429.2x for MI and stroke

### Unadjusted incidence of MI or stroke by age and sex in four cohorts

**Fig. 1:**
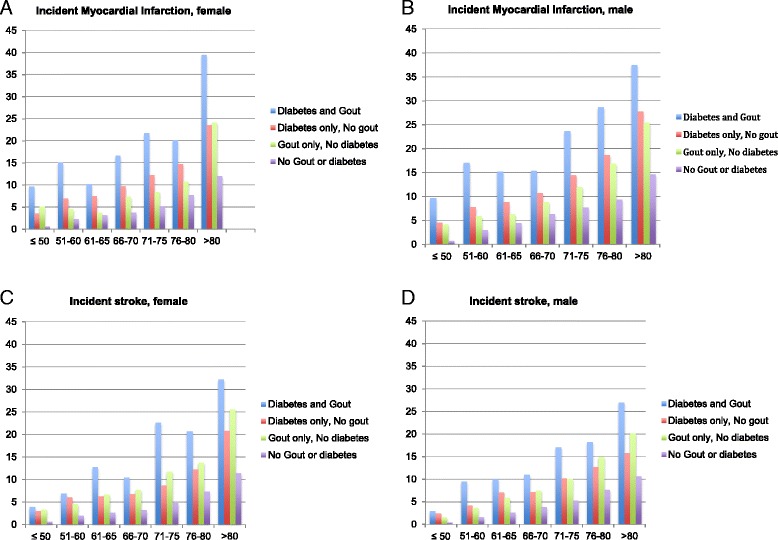
Unadjusted rates/1000 patient-years of incident myocardial infarction (MI) (**a** and **b**) and stroke (**c** and **d**) based on the presence/absence of diabetes and gout, by age and sex. *x*-Axis shows the age groups in years, and *y*-axis shows the incidence rates/1000 patient-years for incident MI in females (**a**) and males (**b**) and of incident stroke in females (**c**) and males (**d**)

Data for the unadjusted incidence of MI (Table 3) and stroke per 1000 patient-years (Table 4) are provided by sex within each age group (*see also* Fig. 1). Unadjusted incidence rates for MI in patients with only gout were similar to or slightly lower than in patients with only diabetes and much higher for patients with both gout and diabetes (Table 3 and Fig. 1a and b). Unadjusted incidence rates for stroke in patients with only gout were similar to or slightly higher than in patients with only diabetes (Table 4 and Fig. 1c and d). Rates for MI and stroke increased with age in all four cohorts (Fig. 1).

**Table 3:** Unadjusted rates of incident myocardial infarction by age and sex based on the presence/absence of diabetes and gout

Group	Females			Males		
Age groups (years)	No. of MI events	Person-years	Incidence rate (/1000 PY)	No. of MI events	Person-years	Incidence rate (/1000 PY)
Diabetes and gout						
≤ 50	5	519.1	9.63	16	1665.0	9.61
51–60	22	1463.6	15.03	45	2642.8	17.03
61–65	14	1376.8	10.17	32	2103.6	15.21
66–70	51	3067.2	16.63	67	4353.8	15.39
71–75	65	2992.6	21.72	83	3507.9	23.66
76–80	51	2531.1	20.15	66	2303.4	28.65
> 80	106	2688.5	39.43	64	1709.0	37.45
Diabetes only, no gout						
≤ 50	113	32,057.2	3.52	125	27,800.2	4.50
51–60	269	38,836.8	6.93	229	29,492.3	7.76
61–65	206	27,596.4	7.46	181	20,494.1	8.83
66–70	439	45,356.6	9.68	360	33,703.8	10.68
71–75	463	37,735.6	12.27	339	23,503.7	14.42
76–80	445	30,209.4	14.73	293	15,681.8	18.68
> 80	696	29,606.4	23.51	317	11,422.9	27.75
Gout only, no diabetes						
≤ 50	6	1177.0	5.10	36	8691.4	4.14
51–60	9	2011.3	4.47	46	7845.4	5.86
61–65	8	2153.1	3.72	37	5882.7	6.29
66–70	43	5861.1	7.34	132	14,896.7	8.86
71–75	53	6308.6	8.40	144	12,016.8	11.98
76–80	70	6510.9	10.75	156	9253.9	16.86
> 80	247	10,232.1	24.14	221	8705.4	25.39
No gout or diabetes						
≤ 50	178	293,997.0	0.61	161	222,722.0	0.72
51–60	277	121,761.0	2.27	228	76,284.1	2.99
61–65	238	76,628.8	3.11	191	43,196.1	4.42
66–70	528	140,311.0	3.76	508	80,566.3	6.31
71–75	614	119,092.0	5.16	485	62,948.0	7.70
76–80	810	105,226.0	7.70	452	48,350.6	9.35
> 80	1738	144,580.0	12.02	684	46,825.3	14.61

*MI* Myocardial infarction, *PY* Person-years

*Baseline International Classification of Diseases, Ninth Revision, Clinical Modification, codes of 410, 412, 430–438, 428.xx, and 429.2X were removed to find incident MI rates

**Table 4:** Unadjusted rates of incident* stroke by age and sex based on the presence/absence of diabetes and gout

Group	Females			Males		
Age groups (years)	No. of stroke events	Person-years	Incidence rate (/1000 PY)	No. of stroke events	Person-years	Incidence rate (/1000 PY)
Diabetes and gout						
≤ 50	2	507.1	3.94	5	1681.4	2.97
51–60	10	1444.4	6.92	25	2629.4	9.51
61–65	17	1329.1	12.79	21	2094.0	10.03
66–70	31	2960.7	10.47	47	4275.3	10.99
71–75	64	2828.7	22.63	58	3392.6	17.10
76–80	50	2412.2	20.73	40	2195.6	18.22
> 80	82	2543.8	32.23	44	1633.0	26.94
Diabetes only, no gout						
≤ 50	95	31,642.3	3.00	66	27,586.3	2.39
51–60	230	37,988.2	6.05	123	29,100.9	4.23
61–65	168	26,836.9	6.26	142	20,078.1	7.07
66–70	295	43,522.1	6.78	235	32,805.9	7.16
71–75	311	35,848.3	8.68	230	22,574.2	10.19
76–80	348	28,436	12.24	189	14,887.7	12.70
> 80	578	27,758.8	20.82	171	10,827.8	15.79
Gout only, no diabetes						
≤ 50	4	1175.3	3.40	14	8647.9	1.62
51–60	9	1964.2	4.58	29	7795.3	3.72
61–65	14	2086.0	6.71	34	5772.2	5.89
66–70	44	5705.1	7.71	110	14,624.7	7.52
71–75	71	6019.3	11.80	117	11,595.8	10.09
76–80	85	6172.5	13.77	132	8866.4	14.89
> 80	247	9655.8	25.58	168	8324.6	20.18
No gout or diabetes						
≤ 50	181	292,332.0	0.62	104	222,180.0	0.47
51–60	235	120,045.0	1.96	121	75,642.6	1.60
61–65	197	75,185.7	2.62	114	42,652.2	2.67
66–70	443	136,150.0	3.25	303	78,992.1	3.84
71–75	549	114,537.0	4.79	326	61,136.1	5.33
76–80	737	100,637.0	7.32	356	46,603.7	7.64
> 80	1581	138,276.0	11.43	479	44,873.5	10.67

*PY* Person-years

*Baseline International Classification of Diseases, Ninth Revision, Clinical Modification, codes of 410, 412, 430–438, 428.xx, and 429.2X were removed to find incident stoke rates

### Gout vs. diabetes as a risk of incident MI or incident stroke

In age-adjusted analyses, compared with diabetes alone, patients with gout alone had a lower risk of MI but a similar risk of stroke, with respective HRs of 0.87 (95% CI 0.81–0.92) and 1.06 (95% CI 0.99–1.14) (Table 5).

**Table 5:** Unadjusted, age-adjusted, and multivariable-adjusted HR of incident myocardial infarction or incident stroke

	Incident MI HR (95% CI) *p* value			Incident stroke HR (95% CI) *p* value		
	Unadjusted	Age-adjusted	Multivariable-adjusted^a^	Unadjusted	Age-adjusted	Multivariable-adjusted^a^
Gout and diabetes	1.89 (1.75–2.05)	1.61 (1.49–1.75)	1.35 (1.25–1.47)	1.91 (1.74–2.10)	1.61 (1.46–1.77)	1.42 (1.29–1.56)
	*p* < 0.0001	*p* < 0.0001	*p* < 0.0001	*p* < 0.0001	*p* < 0.0001	*p* < 0.0001
Gout, no diabetes	1.08 (1.01–1.15)	0.87 (0.81–0.92)	0.81 (0.76–0.87)	1.35 (1.26–1.45)	1.06 (0.99–1.14)	1.02 (0.95–1.10)
	*p* = 0.0187	*p* < 0.0001	*p* < 0.0001	*p* < 0.0001	*p* = 0.1005	*p* = 0.5723
No diabetes, no gout	0.41 (0.39–0.42)	0.45 (0.43–0.46)	0.53 (0.51–0.55)	0.45 (0.43–0.47)	0.50 (0.48–0.52)	0.57 (0.54–0.60)
	*p* < 0.0001	*p* < 0.0001	*p* < 0.0001	*p* < 0.0001	*p* < 0.0001	*p* < 0.0001
Diabetes, no gout	Reference	Reference	Reference	Reference	Reference	Reference

*MI* Myocardial infarction

A nonsignificant HR in age- and multivariable-adjusted models denotes that, with patients with diabetes as the reference category, the HR of incident stroke was similar; in other words, gout is a cerebrovascular risk factor equivalent to diabetes.

Multivariable-adjusted analysis included patients without MI, stroke, and heart disease in the baseline period

^a^Adjusted for age, sex, race, hypertension, hyperlipidemia, chronic obstructive pulmonary disease, chronic kidney disease, and peripheral vascular disease. Additional adjustment for immune diseases led to minimal attenuation of OR but no change in *p* value or interpretation

In multivariable-adjusted analyses, compared with patients with only diabetes, those with only gout had significantly lower HRs of incident MI (HR 0.81, 95% CI 0.76–0.87) but a similar HR of incident stroke (HR 1.02, 95% CI 0.95–1.10) (Table 5). Compared with patients with only diabetes, patients with both gout and diabetes had significantly higher risk for incident MI (HR 1.35, 95% CI 1.25–1.47) and of incident stroke (HR 1.42, 95% CI 1.29–1.56), and those with neither disease had significantly lower HRs of incident MI and incident stroke (Table 5). Conversely, compared with patients without diabetes and gout, higher HRs of incident MI were significantly higher: gout (HR 1.53, 95% CI 1.44–1.63) and both diabetes and gout (HR 2.55, 95% CI 2.35–2.77). Details of the full model, including all covariates, are shown in Additional file 1: Appendix 2. Other significant factors associated with higher HRs for incident MI were older age, male sex, nonwhite race/ethnicity, hyperlipidemia, COPD, and renal disease. Factors significantly associated with incident stroke were similar, except that sex was not significantly associated.

Sensitivity analyses additionally adjusted for use of NSAIDs and antihypertensive drugs revealed minimal change in ORs/HRs and no change in the interpretation of study results (*see* Additional file 1: Appendix 3 for more detail). Sensitivity analyses additionally adjusted for immune diseases, another potential risk factor for MI and stroke owing to associated chronic inflammation, led to minimal attenuation of HR but no change in *p* value or interpretation (data not shown, available from author on request).

## Discussion

Using a large cohort of patients who had gout, diabetes, both, or neither, we investigated whether gout was a risk equivalent for incident MI and stroke, similar to diabetes. We used a systematic approach to address this by analyzing a large U.S. sample and comparing patients with diabetes and gout, patients with only gout, and those with only diabetes. On one hand, our findings indicate that gout was a cerebrovascular risk factor equivalent, similar to diabetes, for stroke. On the other hand, gout was not a cardiovascular risk factor equivalent for incident MI, unlike diabetes. Patients with both gout and diabetes had a 1.4 times multivariable-adjusted risk of incident MI and stroke for each, compared with only diabetes, demonstrating that gout was additive to diabetes in further increasing the risk of stroke and MI, above and beyond the increased risk by diabetes. These novel findings merit further discussion.

To our knowledge, this is the first large observational study to examine gout as a cerebrovascular risk equivalent for stroke equivalent to diabetes. The adjusted HRs for incident stroke were comparable between patients with gout alone and patients with diabetes alone, showing that gout is a cerebrovascular risk equivalent to diabetes. Patients with both diabetes and gout had a 1.4 times multivariable-adjusted HR of incident stroke compared with diabetes alone, further supporting the additive risk of gout to diabetes [15] in patients without underlying coronary artery disease (CAD). However, gout is not a risk equivalent to diabetes for incident MI, an important negative finding in our study. We addressed this important previously raised question of risk equivalency. Not surprisingly, patients with gout and diabetes had higher HRs of MI than those with only diabetes (HR 1.35, 95% CI 1.25–1.47) and those without gout or diabetes (HR 2.55, 95% CI 2.35–2.77). Thus, our study confirms previous findings that gout is an independent risk factor for MI [2–8, 26] and shows that MI risk with gout was additive to the higher risk noted with diabetes. This finding highlights the difference in being a risk factor vs. a risk equivalent to diabetes.

In a recent analysis not adjusted for important cardiovascular risk factors such as hypertension and diabetes compared with the U.K. general population, people with gout had a 1.7 times higher risk of incident stroke [7]. The study included only hospitalized patients with a diagnosis of gout, which might represent a sicker patient subgroup. Thus, the study population, design, and methodology were not comparable to our study. Observational studies adjusted for cardiovascular risk factors showed that gout was associated with 1.26–1.82 times higher odds of MI [6–8] vs. not associated with incident cardiovascular disease [9, 10] vs. associated with MI only in women and not in men [5]. Our study shows that gout was an independent risk factor for incident MI and of incident stroke in patients without underlying CAD.

There are several proposed mechanisms that can mediate the increased stroke risk in patients with gout, such that gout is a stroke risk equivalent of diabetes. Hyperuricemia, the key underlying abnormality in gout, impairs nitric oxide production [27, 28] and activates the renin-angiotensin system [29], leading to endothelial dysfunction [30, 31], which also contributes to increased blood pressure [32, 33]. Persistent systemic and vascular inflammation in gout due to hyperuricemia [34, 35] and monosodium urate crystals [36, 37] may also contribute to atherosclerosis and stimulate prothrombotic activity [38]. Studies show that uric acid stimulates vascular smooth muscle cell proliferation [39–41]. These mechanisms can potentially explain an increased risk of atherosclerosis [42, 43] and a resulting increase in the risk of incident stroke with gout, similar to that seen with diabetes (risk equivalent). This difference in gout being a risk equivalent to diabetes for stroke but not for MI may be due to different pathogenic roles of gout and urate crystals in stroke vs. MI. Gout also increases the risk of atrial fibrillation [44, 45], a risk factor for stroke but not MI, which may also increase the difference. Systemic inflammation associated with gout may have a differential effect on the risk of incident MI vs. stroke. In this database study, we did not perform any laboratory assays that could provide more insight into underlying mechanisms of acute MI or stroke. Future prospective studies can address this knowledge gap. Despite a few similar risk factors (sex, age, hypertension, diabetes, smoking, obesity, dyslipidemia), other risk factors for stroke (atrial fibrillation, alcohol use) differ from those for MI (modest alcohol use reduces risk) as well as pathogenesis [46–48].

The clinical implications of these study findings may be significant. Although our findings need confirmation in future studies, our recognition of gout as a cerebrovascular risk equivalent to diabetes implies that primary, secondary, and tertiary prevention of stroke may be needed in patients with gout to reduce this increased risk of stroke. On the basis of these findings, screening and aggressive treatment of risk factors for stroke (hypertension, diabetes, hyperlipidemia) may be warranted in patients with gout. It remains to be seen whether physicians may also consider empiric use of lipid-lowering agents in a subgroup of patients with gout at high risk of cardiovascular events, similar to an approach in diabetes, but this needs further study. Optimal treatment of gout targeting both hyperuricemia and systematic inflammation may also be needed to reduce the risk of stroke in patients with gout. What remains to be seen is to what extent each of these strategies reduces the risk of stroke in patients with gout. Large-sample observational studies and/or randomized trials in high-risk gout patients can answer this question in the future. These study findings indicate that cardiovascular disease prevention and treatment strategies that are in place for patients with diabetes [15] may also be applicable to patients with gout.

Gout has been linked to higher overall mortality in previous studies [3, 4, 49], including higher cardiovascular mortality [3, 50]. MI and stroke are major contributors to cardiovascular mortality and to overall mortality. Our study provides further insight and a further rationale for this previous finding by showing that gout is a cerebrovascular risk equivalent of diabetes for stroke, which is associated with high cardiovascular mortality. Authors of a recent review explained the links of uric acid to cardiovascular disease by examining its associations with oxidative stress, inflammation, and immune activation [51].

Several limitations must be considered while interpreting the findings of our study. Smoking status and lipid levels (including high-density lipoprotein) were not available in our database, and therefore we could not adjust for these Framingham risk factors in our analyses. We used a diagnostic code for hypercholesterolemia or the use of statins to capture patients with hypercholesterolemia, which may still have led to underdiagnosis owing to some patients not wanting/taking statins for hypercholesterolemia and owing to undercoding. Other potential risk factors for MI/stroke, including body mass index and alcohol use, were not available in the database; therefore, we were not able to adjust for these factors in the analyses. Serum urate levels were available for a very small proportion of patients, not allowing us to incorporate this in mediation analysis in this database study. Therefore, we could not examine whether the associations were mediated via hyperuricemia and whether the effects differed by the level of serum urate.

Another study limitation was that the study period of 2007–2010 resulted in a short mean follow-up period; however, we had 1.34 million observed study periods, which provided adequate study power. As in most commercial claims datasets, death was not available as a variable, aside from in-hospital mortality, which was not sufficient to run a competing risk analysis. We used prevalent diabetes and gout rather than incident gout or diabetes to have enough cases of outcomes of interest, incident stroke or MI, a key study consideration in this analysis. Researchers in future studies should consider using incident disease instead of prevalent disease. Positive predictive value for gout diagnosis was based on a study done at a Veterans Affairs (VA) medical center [18], and it is possible that coding in the Medicare system may be either more or less accurate than in the VA system. The very high prevalence of hyperlipidemia in patients with diabetes and the high prevalence in other populations (gout, neither) in our study are similar to the reported prevalence in the U.S. general population and in subjects with diabetes [52, 53]; inclusion of the use of statins in the definition may have increased the sensitivity of the definition. Study strengths include a large sample size and the availability of a cohort with diabetes, allowing comparison to a risk equivalent, robust estimates tested in multiple sensitivity analyses, and use of a population without baseline heart disease.

## Conclusions

We found that, compared with diabetes, gout was a risk equivalent for incident stroke but not a risk equivalent for incident MI. Gout was additive to diabetes in increasing the risk of both incident MI and incident stroke. As an inflammatory disease commonly associated with high comorbidity load (hypertension, diabetes, hyperlipidemia, obesity, renal failure) and systemic inflammation, gout can be effectively treated with inexpensive generic medications. On the basis of our study results, the presence of gout should alert physicians to screen, diagnose, and promptly treat cardiovascular risk factors (hypertension, hyperlipidemia), similar to the approach used in patients with diabetes. The increased risk of stroke may prompt patients and physicians to consider empiric lipid-lowering treatment in at least those patients with gout who have high cardiovascular risk and might be predisposed to poor outcomes. Researchers in future studies need to examine the strategies for such as approach and estimate its effect on stroke and other cardiovascular outcomes in patients with gout.

## Additional file

Additional file 1:
**Appendix 1.** ICD-9-CM diagnostic codes for each condition used for outcome, cohort eligibility, and covariate definitions. The file provides a list of all ICD-9-CM diagnostic codes for the study outcomes (incident stroke, incident myocardial infarction), disease cohorts (gout, diabetes), baseline covariates (hypertension, chronic obstructive pulmonary disease, chronic kidney disease, statin use, hyperlipidemia, autoimmune disease), and codes for baseline exclusion for prevalent stroke or myocardial infarction, and censoring rules. **Appendix 2.** Full multivariable-adjusted models for the risk of incident MI and incident stroke (as shown in Table 5 multivariable models) with all included covariates. This file shows the complete listing of all the variables from the final full multivariable-adjusted models for the risk of incident MI and incident stroke. **Appendix 3.** Sensitivity analyses for the main multivariable-adjusted model, adjusting additionally for antihypertensive drugs and nonsteroidal anti-inflammatory drugs. (DOCX 30 kb)

## Abbreviations

CAD: Coronary artery disease
CHD: Coronary heart disease
COPD: Chronic obstructive pulmonary disease
DM: Diabetes mellitus
ICD-9-CM: International Classification of Diseases, Ninth Revision, Clinical Modification
MI: Myocardial infarction
MPCD: Multi-Payer Claims Database
NSAID: Nonsteroidal anti-inflammatory drug
PVD: Peripheral vascular disease
STROBE: Strengthening the Reporting of Observational Studies in Epidemiology
VA: Veterans Affairs
