# Supplementary material for: Is gout a risk equivalent to diabetes for stroke and myocardial infarction? A retrospective claims database study
Source: Arthritis Res Ther. 2017 Oct 17;19:228. doi: 10.1186/s13075-017-1427-5 (PMC5646136; doi:10.1186/s13075-017-1427-5)
Supplement: Additional file 1: — Appendix 1. ICD-9-CM diagnostic codes for each condition used for outcome, cohort eligibility, and covariate definitions. The file provides a list of all ICD-9-CM diagnostic codes for the study outcomes (incident stroke, incident myocardial infarction), disease cohorts (gout, diabetes), baseline covariates (hypertension, chronic obstructive pulmonary disease, chronic kidney disease, statin use, hyperlipidemia, autoimmune disease), and codes for baseline exclusion for prevalent stroke or myocardial infarction, and censoring rules. Appendix 2. Full multivariable-adjusted models for the risk of incident MI and incident stroke (as shown in Table 5 multivariable models) with all included covariates. This file shows the complete listing of all the variables from the final full multivariable-adjusted models for the risk of incident MI and incident stroke. Appendix 3. Sensitivity analyses for the main multivariable-adjusted model, adjusting additionally for antihypertensive drugs and nonsteroidal anti-inflammatory drugs. (DOCX 30 kb) [file 13075_2017_1427_MOESM1_ESM.docx]

**Additional file 1**

**Appendix 1. ICD-9-CM Diagnostic Codes for each condition used for outcome, cohort eligibility, and covariate definitions**

|  | ICD-9-CM Codes |
| --- | --- |
| Study Outcome |  |
| Incident myocardial infarction (MI) | 410.x1: 410.01, 410.11, 410.21, 410.31, 410.41, 410.51, 410.61, 410.71, 410.81, 410.91 |
| Incident stroke | 430.xx: all codes with 430 in the first 3 places  431.xx: all codes with 431 in the first 3 places  433.x1: 433.01, 433.11, 433.21, 433.31, 433.41, 433.51, 433.61, 433.71, 433.81, 433.91  434.xx except 434.x0: all codes with 434 in the first 3 places except the ones with 0 in the last place  436.xx: all codes with 436 in the first 3 places |
|  |  |
| Disease cohorts |  |
| Gout | 274.xx |
| Diabetes | 250.xx |
|  |  |
| Baseline covariates |  |
| Hypertension | 401.xx - 405.xx |
| Chronic obstructive pulmonary disease (COPD) | 491.xx, 492.xx, 496.xx |
| Chronic kidney disease | 582.xx, 583.xx, 585.xx, 586.xx, 588.xx |
| Peripheral vascular disease (PVD) | 440.20-440.24, 440.31-440.32, 440.8, 440.9, 443.9, 785.4, V434, 441.xx |
| Statin use | No code; from medication fill data |
| Hyperlipidemia | 272.0, 272.1, 272.2, 272.3, 272.4 |
| Autoimmune disease | 720.xx, 283.xx, 579.xx, 255.xx, 242.xx, 357.xx, 555.xx, 556.xx, 359.xx, 340.xx, 358.xx, 281.xx, 725.xx, 696.xx, 710.xx, 255.4x, 279.8x, 694.5x, 710.3x, 245.2x, 695.4x, 694.4x, 694.5x, 694.6x, 710.4x, 571.6, 696.1, 410.2x, 446.5x, 446.4x, 323.61, 571.42, 446.21, 287.31, 258.01, 362.18 |
|  |  |
| Baseline exclusion | MI [410.xx or 412.xx], stroke [430-438] or heart disease [410-414, 428.xx and 429.2x] |
|  |  |
| Censoring (includes prevalent cases and outpatient codes) |  |
| Myocardial infarction (MI) | An outpatient code of 410.x1 or an inpatient or outpatient code of 410 other than 410.x1 and 412 |
| Stroke | Outpatient code for stroke (430.xx, 431.xx, 433.x1, 436.xx, 434.xx except 434.x0) or an inpatient or outpatient code of 430-438 other than that of stroke ICD-9- codes listed at the beginning of the sentence. |

**Appendix 2. Full multivariable-adjusted models for the risk of Incident MI and incident stroke (as shown in Table 5 multivariable models) with all included covariates**

|  | Incident MI | | Incident stroke | |
| --- | --- | --- | --- | --- |
|  | **Hazard ratio**  **(95% CI)** | **p-value** | **Hazard ratio**  **(95% CI)** | **p-value** |
| Gout and diabetes | 1.35  (1.25, 1.47) | <0.0001 | 1.42  (1.29, 1.56) | <0.0001 |
| Gout, no diabetes | 0.81  (0.76, 0.87) | <0.0001 | **1.02**  **(0.95, 1.10)** | **0.5723** |
| No diabetes, no gout | 0.53  (0.51, 0.55) | <0.0001 | 0.57  (0.54, 0.60) | <0.0001 |
| Diabetes, no gout | Ref |  | Ref |  |
| Gender |  |  |  |  |
| Male | 1.26  (1.22, 1.30) | <0.0001 | 1.00  (0.96, 1.04) | 0.9994 |
| Female | Ref |  | Ref |  |
| Age group |  |  |  |  |
| ≤50 | 0.35  (0.31, 0.38) | <0.0001 | 0.30  (0.26, 0.34) | <0.0001 |
| 51-60 | 0.90  (0.82, 0.98) | 0.0182 | 0.77  (0.70, 0.85) | <0.0001 |
| 66-70 | 1.23  (1.13, 1.33) | <0.0001 | 1.13  (1.04, 1.24) | 0.0063 |
| 71-75 | 1.57  (1.45, 1.70) | <0.0001 | 1.58  (1.45, 1.73) | <0.0001 |
| 76-80 | 2.02  (1.87, 2.18) | <0.0001 | 2.19  (2.01, 2.39) | <0.0001 |
| >80 | 3.15  (2.93, 3.39) | <0.0001 | 3.29  (3.03, 3.57) | <0.0001 |
| 61-65 | Ref |  | Ref |  |
| Race |  |  |  |  |
| Asian | 0.62  (0.55, 0.70) | <0.0001 | 0.71  (0.62, 0.80) | <0.0001 |
| Black | 0.92  (0.87, 0.97) | 0.0020 | 1.36  (1.29, 1.44) | <0.0001 |
| Hispanic | 0.64  (0.58, 0.70) | <0.0001 | 0.77  (0.69, 0.85) | <0.0001 |
| Other | 0.81  (0.70, 0.95) | 0.0072 | 1.09  (0.93, 1.27) | 0.3096 |
| Missing | 0.29  (0.26, 0.32) | <0.0001 | 0.33  (0.29, 0.36) | <0.0001 |
| White | Ref |  | Ref |  |
| Comorbidities |  |  |  |  |
| Hypertension | 1.16  (1.11, 1.20) | <0.0001 | 1.37  (1.31, 1.44) | <0.0001 |
| COPD | 1.61  (1.53, 1.69) | <0.0001 | 1.29  (1.21, 1.37) | <0.0001 |
| Renal disease | 1.79  (1.69, 1.88) | <0.0001 | 1.44  (1.35, 1.53) | <0.0001 |
| Hyperlipidemia | 0.98  (0.94, 1.02) | 0.2938 | 0.82  (0.78, 0.85) | <0.0001 |
| PVD | 1.67  (1.58, 1.77) | <0.0001 | 1.40  (1.30, 1.50) | <0.0001 |

**Appendix 3. Sensitivity analyses for the main multivariable-adjusted model, adjusting additionally for anti-hypertensive drugs* and non-steroidal anti-inflammatory drugs**

|  | Incident MI | | Incident stroke |  |  |
| --- | --- | --- | --- | --- | --- |
|  | **Hazard ratio**  **(95% CI)** | **p-value** | **Hazard ratio**  **(95% CI)** | **p-value** | |
| Gout and diabetes | 1.16 (1.06, 1.26) | 0.0007 | 1.25 (1.13, 1.38) | <0.0001 | |
| Gout, no diabetes | 0.73 (0.68, 0.78) | <0.0001 | **0.94 (0.87, 1.01)** | **0.08** | |
| No diabetes, no gout | 0.50 (0.48, 0.52) | <0.0001 | 0.54 (0.52, 0.57) | <0.0001 | |
| Diabetes, no gout | Ref | Ref | Ref |  | |

*Anti-hypertensive use was defined as the use of any of the following medications at baseline

- ACE inhibitors (benazepril, captopril, enalapril, fosinopril, lisinopril, moexipril, perindopril, quinapril, ramipril, trandolapril)
- Calcium channel blockers (amlodipine, bepridil, clevidipine, diltiazem, felodipine, isradipine, mibefradil, nicardipine, nifedipine, nimodipine, nisoldipine, verapamil)
- Thiazide diuretics (bendroflumethiazide, chlorothiazide, chlorthalidone, hydrochlorothiazide, hydroflumethiazide, indapamide, methyclothiazide, metolazone, polythiazide, trichlormethiazide, hydrochlorthiazide)
- Potassium-sparing diuretics (amiloride, triamterene)
- Loop diuretics (bumetanide, ethacrynate, ethacrynic acid, furosemide, torsemide)
- Aldosterone-antagonist (eplerenone, spironolactone)
- Alpha-blockers (doxazosin, prazosin, terazosin)
- Angiotensin-receptor blockers(azilsartan, candesartan, eprosartan, irbesartan, losartan, olmesartan, telmisartan, valsartan)
- Direct renin inhibitor: aliskiren
- Beta-blockers (acebutolol, atenolol, betaxolol, bisoprolol, carteolol, carvedilol, esmolol, labetalol, metoprolol, nadolol, nebivolol, penbutolol,
- pindolol, propranolol, sotalol, timolol;
- Central-acting (clonidine, guanabenz, guanfacine, methyldopa, methyldopate)
- Vasodilators (diazoxide, hydralazine, minoxidil, nitroprusside sodium, tolazoline)
